# Comparative discriminative ability of CVAI and traditional insulin resistance indices for MAFLD in Chinese adults with type 2 diabetes

**DOI:** 10.3389/fendo.2026.1839268

**Published:** 2026-07-03

**Authors:** Jian Yang, Fanci Xie, Xiaoli Zhu, Hairong Zhou

**Affiliations:** 1 Department of General Medicine, Longhua District People’s Hospital, Shenzhen, China; 2 The Fourth Clinical Medical College of Guangzhou University of Chinese Medicine, Shenzhen, China; 3 Department of Emergency Medicine, Shenzhen Second People’s Hospital, Shenzhen, China

**Keywords:** CVAI, discriminative value, insulin resistance, MAFLD, type 2 diabetes mellitus

## Abstract

**Background:**

Metabolic dysfunction-associated fatty liver disease (MAFLD) is highly prevalent in patients with type 2 diabetes mellitus (T2DM), necessitating simple and reliable non-invasive screening tools. The Chinese Visceral Adiposity Index (CVAI) has been developed to assess visceral adipose dysfunction, but its discriminative performance for MAFLD compared with traditional insulin resistance (IR) indices in T2DM populations remains unclear.

**Methods:**

This retrospective cross-sectional study included 2,945 Chinese adults with T2DM recruited from 45 community health centers. We calculated seven IR indices: CVAI, TyG, TyG-BMI, TyG-WC, TyG-WHtR, METS-IR, and SPISE. Multivariable logistic regression was employed to assess independent associations with MAFLD. Discriminative ability was evaluated using receiver operating characteristic (ROC) curve analysis with Delong’s tests. Net reclassification improvement (NRI) and integrated discrimination improvement (IDI) were calculated to assess incremental discriminative value over a baseline clinical model. Restricted cubic spline (RCS) regression, decision curve analysis (DCA), and subgroup analyses were also performed.

**Results:**

Among participants, 1,313 (44.6%) were diagnosed with MAFLD. All IR indices were independently associated with MAFLD after full adjustment (all P < 0.001). CVAI demonstrated the highest discriminative ability (AUC = 0.754, 95% CI: 0.737–0.771), significantly outperforming all other indices (all P < 0.001). Adding CVAI to the baseline model significantly improved risk reclassification (NRI = 0.598, 95% CI: 0.533–0.665; IDI = 0.081, 95% CI: 0.072–0.090) and yielded the broadest clinical utility across threshold probabilities of 10–90% in DCA. The association between CVAI and MAFLD was significantly stronger in females and older adults (both P for interaction < 0.05). RCS analysis revealed a predominantly linear relationship (non-linear P = 0.0581).

**Conclusion:**

CVAI is independently associated with MAFLD and exhibits superior discriminative performance compared with traditional IR indices in Chinese adults with T2DM. As a simple, non-invasive index derived from routine clinical parameters, CVAI holds promise as an effective screening tool for MAFLD, particularly in females and older adults. Prospective studies are warranted to validate its diagnostic utility.

## 1 Introduction

Metabolic dysfunction-associated fatty liver disease (MAFLD) has emerged as the most prevalent chronic liver condition worldwide, affecting approximately 30–40% of the global adult population (1). The disease spectrum encompasses simple hepatic steatosis, metabolic dysfunction-associated steatohepatitis (MASH), and can progress to cirrhosis and hepatocellular carcinoma (2, 3). Importantly, MAFLD is not confined to the liver; it is a multisystem disorder intricately linked with an increased risk of type 2 diabetes mellitus (T2DM), cardiovascular disease, and chronic kidney disease (4–6). In China, rapid urbanization and sedentary lifestyles have led to a MAFLD prevalence of approximately 29.7%, imposing a substantial public health burden (7, 8).

The bidirectional relationship between T2DM and MAFLD is particularly concerning. T2DM, characterized by chronic hyperglycemia and insulin resistance (IR), affects over 140 million adults in China (9). IR serves as the common pathophysiological basis for both conditions, promoting increased lipolysis, accumulation of free fatty acids in the liver, and hepatic gluconeogenesis, thereby creating a vicious cycle (10, 11). Epidemiological data indicate that the prevalence of MAFLD in patients with T2DM is alarmingly high, ranging from 60% to 70%, with a significant proportion exhibiting advanced fibrosis (12, 13). This coexistence exacerbates the risk of both microvascular and macrovascular complications, underscoring the urgent need for early and accurate identification of high-risk individuals within this population (14).

Although liver biopsy remains the gold standard for diagnosing and staging MASH and fibrosis, its invasiveness, potential for sampling error, and poor patient acceptance render it unsuitable for widespread screening or routine clinical practice, especially in high-prevalence populations such as those with T2DM (15, 16). Consequently, there is a pressing need for simple, non-invasive, and cost-effective biomarkers to identify T2DM patients at high risk for MAFLD. Given that IR is a core driver of MAFLD pathogenesis, several surrogate indices derived from routine biochemical measurements and anthropometric parameters have been developed (17). The triglyceride-glucose (TyG) index and its combination with obesity indicators (e.g., TyG-BMI, TyG-WC) have been widely validated as reliable markers of IR and have shown discriminative value for cardiovascular disease and MAFLD (18–20). The METS-IR index, which integrates TG, HDL-C, BMI, and blood glucose, has also been proposed as a simple tool for NAFLD risk assessment in non-obese populations (21). However, the comparative performance of these indices for diagnosing MAFLD in the unique metabolic milieu of Chinese T2DM patients remains to be fully elucidated.

More recently, attention has shifted toward the crucial role of visceral adipose tissue dysfunction. Unlike general obesity, visceral adiposity is more strongly linked to IR, systemic inflammation, and the progression of fatty liver disease (22, 23). To specifically address this in the Chinese population, the Chinese Visceral Adiposity Index (CVAI) was developed and validated. CVAI incorporates age, BMI, waist circumference, and lipid profiles (TG and HDL-C) to provide a reliable estimate of visceral fat function (24). Accumulating evidence has demonstrated that CVAI is superior to traditional anthropometric measures in predicting the incidence of prediabetes, T2DM, and metabolic syndrome in Chinese adults (25, 26). Furthermore, a cross-sectional study suggested a positive correlation between CVAI and the risk of MAFLD in the general Chinese population (27).

However, despite the mechanistic link between visceral adiposity and MAFLD, evidence specifically examining the association between CVAI and MAFLD in patients with established T2DM is limited. Moreover, it remains unclear whether CVAI, as a marker of visceral fat dysfunction, offers superior diagnostic ability for MAFLD compared with more traditional, glucose-centric IR indices like TyG or composite scores like METS-IR in this high-risk diabetic cohort. Therefore, the current study aimed to comprehensively evaluate and compare the discriminative ability of CVAI, TyG, TyG-BMI, TyG-WC, TyG-WHtR, METS-IR, and SPISE for the presence of MAFLD in a large community-based cohort of Chinese adults with type 2 diabetes.

## 2 Materials and methods

### 2.1 Study design and population

We conducted a retrospective cross-sectional analysis using de-identified electronic health records from a network of 45 community health centers affiliated with Longhua District People’s Hospital of Shenzhen. The investigation included 2,945 adults diagnosed with type 2 diabetes mellitus who received medical services between 2023 and 2024.

Eligibility criteria required participants to be: (1) aged 18 years or older, and (2) diagnosed with T2DM according to American Diabetes Association standards (28). Exclusion criteria encompassed: (1) diagnosis of type 1 diabetes, gestational diabetes, or other specific diabetes types; (2) other causes of fatty liver disease (including significant alcohol consumption defined as ≥210 g/week for men and ≥140 g/week for women, viral hepatitis, drug-induced liver disease, or autoimmune liver disease); (3) severe systemic conditions such as heart failure (NYHA Class III/IV), advanced liver cirrhosis, autoimmune diseases, hematological malignancies, or active cancer; (4) active infectious diseases, systemic inflammatory conditions, or those on immunosuppressants; and (5) incomplete data necessary for the calculation of insulin resistance indices or MAFLD diagnosis.

### 2.2 Ethics statement

The study protocol was reviewed and approved by the Ethics Committee of Longhua District People’s Hospital (Approval No.: 2026.03; Approval Date: January 2026). All data handling complied with institutional security protocols and relevant data protection regulations. The research adhered fully to the ethical principles outlined in the Declaration of Helsinki and relevant international guidelines governing human subjects research and retrospective data analysis. The ethics committee waived the requirement for individual informed consent due to the use of de-identified retrospective data.

### 2.3 Data collection and variable definitions

The electronic health records provided information across three domains:

Demographics and lifestyle history: Age, sex (0 = Female, 1 = Male), smoking status (0 = No, 1 = Yes), alcohol consumption (1 = Never, 2 = Occasionally, 3 = Frequently), physical activity (1 = No exercise, 2 = <3 times/week, 3 = ≥3 times/week), and education level (1 = Basic, 2 = Intermediate, 3 = Advanced).

Clinical history: Hypertension (0 = No, 1 = Yes), cerebrovascular disease (0 = No, 1 = Yes), and heart disease (0 = No, 1 = Yes).

Anthropometric and laboratory parameters: Body mass index (BMI) calculated as weight in kilograms divided by height in meters squared (kg/m²); systolic blood pressure (SBP); diastolic blood pressure (DBP); glycated hemoglobin (HbA1c); alanine aminotransferase (ALT); aspartate aminotransferase (AST); total bilirubin (TBIL); triglycerides (TG); total cholesterol (TC); low-density lipoprotein cholesterol (LDL-C); high-density lipoprotein cholesterol (HDL-C); fasting plasma glucose (FPG); serum creatinine (Cr); blood urea nitrogen (BUN); uric acid (UA); hemoglobin (HGB); white blood cell count (WBC); platelet count (PLT); and urinary albumin-to-creatinine ratio (uACR).

### 2.4 Definition of insulin resistance indices

We calculated the following insulin resistance indices based on previously published formulas:

TyG index = Ln [TG (mg/dL) × FPG (mg/dL)/2] (19).

TyG-BMI = TyG index × BMI (19).

TyG-WC = TyG index × waist circumference (cm) (19).

TyG-WHtR = TyG index × (waist circumference/height) (29).

CVAI (Chinese Visceral Adiposity Index):

For males: CVAI = −267.93 + 0.68 × age (years) + 0.03 × BMI (kg/m²) + 4.00 × waist circumference (cm) + 22.00 × log10(TG) − 16.32 × HDL-C (mmol/L) (30).

For females: CVAI = −187.32 + 1.71 × age (years) + 4.23 × BMI (kg/m²) + 1.12 × waist circumference (cm) + 39.76 × log10(TG) − 11.66 × HDL-C (mmol/L) (30).

METS-IR (Metabolic Score for Insulin Resistance) = ln(2 × FPG(mg/dL) + TG(mg/dL)) × BMI/ln(HDL-C(mg/dL)) (21).

SPISE (Single-Point Insulin Sensitivity Estimator) = 600 × HDL-C(mg/dL)^0.185/(TG(mg/dL)^0.2 × BMI^1.338) (31).

For indices requiring unit conversion (TyG, METS-IR, and SPISE), TG, HDL-C, and FPG values were converted from mmol/L to mg/dL using standard conversion factors (TG: 1 mmol/L = 88.57 mg/dL; HDL-C: 1 mmol/L = 38.67 mg/dL; FPG: 1 mmol/L = 18 mg/dL).

### 2.5 Outcome definition

The primary outcome was the presence of metabolic dysfunction-associated fatty liver disease (MAFLD). MAFLD was diagnosed based on abdominal ultrasound evidence of hepatic steatosis, in conjunction with the presence of at least one of the following metabolic abnormalities (32): (1) overweight or obesity (BMI ≥24 kg/m²), (2) type 2 diabetes mellitus, or (3) evidence of metabolic dysregulation (including hypertension, hypertriglyceridemia, low HDL-C, or insulin resistance). Participants meeting these criteria were categorized into the MAFLD group (y = 1), while those without hepatic steatosis were categorized into the non-MAFLD group (y = 0).

### 2.6 Statistical analysis

All statistical analyses were performed using R software (version 4.3.1; R Foundation for Statistical Computing). A two-sided P value < 0.05 indicated statistical significance. Normality of continuous variables was assessed using the Shapiro–Wilk test.

Baseline characteristics: For continuous variables, normally distributed data are presented as mean ± standard deviation and compared using Student’s *t*-test, while non-normally distributed data are reported as median with interquartile range and compared using the Mann–Whitney U test. Categorical variables are expressed as frequencies (percentages) and compared via chi-square tests.

Multivariable logistic regression: To quantify the independent associations between each insulin resistance index and MAFLD, we employed multivariable logistic regression with three sequentially adjusted models:

Model 1: adjusted for basic demographics (age and sex);

Model 2: further adjusted for lifestyle factors and comorbidities (smoking, alcohol consumption, physical activity, hypertension, cerebrovascular disease, heart disease, and education);

Model 3 (fully adjusted): additionally adjusted for metabolic and laboratory parameters (HbA1c, ALT, uACR, TC, LDL).

Due to the retrospective nature of the data, certain lifestyle and dietary variables (e.g., detailed dietary composition, fructose intake, sleep quality/duration, and medication history including statins or steroids) were not available and therefore could not be adjusted for, which we acknowledge as a limitation. For each insulin resistance index, odds ratios (ORs) with corresponding 95% confidence intervals (CIs) were extracted and summarized.

ROC curve analysis: We evaluated the discriminative ability of each insulin resistance index for MAFLD using receiver operating characteristic (ROC) curve analysis. The area under the ROC curve (AUC) with 95% CI was calculated for each index. Optimal cut-off values were determined using the Youden index, and corresponding sensitivity and specificity were reported. Pairwise comparisons of AUCs between CVAI and each of the other indices were performed using DeLong’s test.

Reclassification improvement: To assess the incremental discriminative value of adding insulin resistance indices to a baseline clinical model for MAFLD, we calculated continuous net reclassification improvement (NRI) and integrated discrimination improvement (IDI). The baseline model included age, sex, smoking status, alcohol consumption, physical activity, and hypertension. We evaluated three indices (CVAI, TyG, and SPISE) individually against the baseline model. Furthermore, to directly compare the performance of CVAI with that of TyG and SPISE, we also calculated NRI and IDI for the comparisons of Base+CVAI vs. Base+TyG and Base+CVAI vs. Base+SPISE. To obtain robust 95% CIs, bootstrap resampling with 1000 iterations was performed. Statistical significance was determined by the proportion of bootstrap estimates with values below or above zero, using a two-tailed P < 0.05 threshold.

Decision curve analysis (DCA): To evaluate the clinical utility of models incorporating different insulin resistance indices, we performed decision curve analysis. Net benefits were calculated across a range of threshold probabilities for four models: baseline model, baseline + CVAI, baseline + TyG, and baseline + SPISE.

Restricted cubic spline (RCS) analysis: We explored potential non-linear relationships between CVAI and MAFLD risk by fitting multivariable logistic regression models incorporating restricted cubic splines with four knots placed at the 5th, 35th, 65th, and 95th percentiles of the CVAI distribution. The overall association and non-linearity were assessed using likelihood ratio tests. The reference value was set at the median CVAI level.

Subgroup analysis and interaction: To evaluate the consistency of the association between CVAI and MAFLD across different patient strata, we conducted subgroup analyses based on sex (female vs. male), age (<65 vs. ≥65 years), hypertension status (no vs. yes), BMI (<25 vs. ≥25 kg/m²), and HbA1c (<7% vs. ≥7%). Within each subgroup, we estimated the OR for MAFLD per unit increase in CVAI using fully adjusted models. Interaction terms between CVAI and subgroup variables were introduced into the models, and likelihood ratio tests were used to determine the significance of interactions. Results were visualized using a forest plot.

## 3 Results

### 3.1 Baseline characteristics of the study population

A total of 2,945 participants with type 2 diabetes mellitus were included, of whom 1,313 (44.6%) were diagnosed with MAFLD. Baseline characteristics stratified by MAFLD status are summarized in **Table 1**.

**Table 1:** Baseline Characteristics by MAFLD Status.

Variable	Overall (N = 2945)^1^	on-MAFLD (N = 1632)^1^	MAFLD (N = 1313)^1^	P-value^2^
Gender				<0.001
Female	1,414 (48%)	689 (42%)	725 (55%)	
Male	1,531 (52%)	943 (58%)	588 (45%)	
Age (years)	64.02 ± 11.72	59.18 ± 11.51	70.03 ± 8.85	<0.001
Education				<0.001
Basic	1,832 (62%)	911 (56%)	921 (70%)	
Intermediate	749 (25%)	480 (29%)	269 (20%)	
Advanced	364 (12%)	241 (15%)	123 (9.4%)	
Physical				<0.001
No exercise	831 (28%)	482 (30%)	349 (27%)	
<3 times/week	356 (12%)	251 (15%)	105 (8.0%)	
≥3 times/week	1,758 (60%)	899 (55%)	859 (65%)	
Drinking				<0.001
Never	2,183 (74%)	1,154 (71%)	1,029 (78%)	
Occasionally	482 (16%)	323 (20%)	159 (12%)	
Frequently	280 (9.5%)	155 (9.5%)	125 (9.5%)	
Smoking	908 (31%)	560 (34%)	348 (27%)	<0.001
Hypertension	750 (25%)	265 (16%)	485 (37%)	<0.001
Cerebrovascular	153 (5.2%)	52 (3.2%)	101 (7.7%)	<0.001
Heart_disease	262 (8.9%)	109 (6.7%)	153 (12%)	<0.001
Height (cm)	161.02 ± 8.64	161.97 ± 8.47	159.85 ± 8.71	<0.001
Weight (kg)	65.21 ± 11.04	64.53 ± 11.47	66.05 ± 10.42	<0.001
Waist (cm)	88.60 ± 8.80	86.54 ± 8.76	91.17 ± 8.16	<0.001
HGB (g/L)	139.40 ± 16.79	140.07 ± 17.34	138.57 ± 16.04	0.015
WBC (×10⁹/L)	6.60 ± 1.74	6.58 ± 1.80	6.62 ± 1.66	0.488
PLT (×10⁹/L)	235.51 ± 64.90	235.77 ± 65.81	235.20 ± 63.77	0.812
FPG (mmol/L)	8.33 ± 2.90	8.13 ± 2.92	8.58 ± 2.85	<0.001
HbA1c (%)	7.71 ± 1.82	7.56 ± 1.87	7.90 ± 1.74	<0.001
ALT (U/L)	25.83 ± 20.58	26.08 ± 19.22	25.51 ± 22.14	0.464
AST (U/L)	24.08 ± 16.02	24.14 ± 17.37	24.00 ± 14.18	0.806
TBIL (μmol/L)	13.15 ± 6.60	13.43 ± 6.63	12.81 ± 6.56	0.012
Cr (μmol/L)	78.63 ± 43.46	79.08 ± 50.50	78.07 ± 32.68	0.512
BUN (mmol/L)	6.21 ± 3.16	6.18 ± 2.84	6.24 ± 3.51	0.656
TC (mmol/L)	5.01 ± 1.29	5.00 ± 1.34	5.03 ± 1.23	0.602
TG (mmol/L)	2.08 ± 1.84	2.00 ± 1.96	2.19 ± 1.66	0.004
LDL (mmol/L)	3.07 ± 1.04	3.06 ± 1.10	3.09 ± 0.96	0.585
HDL (mmol/L)	1.27 ± 0.36	1.29 ± 0.38	1.24 ± 0.32	<0.001
uACR (mg/g)	136.61 ± 671.01	119.46 ± 463.12	157.92 ± 861.91	0.145
SBP (mmHg)	135.56 ± 17.06	132.46 ± 16.93	139.42 ± 16.43	<0.001
DBP (mmHg)	81.55 ± 10.20	81.60 ± 10.57	81.47 ± 9.72	0.722
BMI(kg/m²)	25.08 ± 3.29	24.50 ± 3.36	25.79 ± 3.05	<0.001
TyG	9.28 ± 0.73	9.19 ± 0.75	9.39 ± 0.68	<0.001
TyG_BMI	233.08 ± 38.23	225.73 ± 39.75	242.21 ± 34.11	<0.001
TyG_WC	823.19 ± 112.24	796.52 ± 113.92	856.34 ± 100.76	<0.001
TyG_WHtR	5.12 ± 0.71	4.92 ± 0.69	5.37 ± 0.65	<0.001
CVAI	122.10 ± 35.79	108.46 ± 34.20	139.02 ± 30.02	<0.001
SPISE	6.29 ± 1.57	6.61 ± 1.73	5.88 ± 1.23	<0.001
METS_IR	40.09 ± 7.29	38.84 ± 7.39	41.64 ± 6.85	<0.001

^1^n (%); Mean ± SD.

^2^Pearson's Chi-squared test; Welch Two Sample t-test.

Compared with non-MAFLD individuals, those with MAFLD were significantly older and had a higher proportion of females (both P < 0.001). The MAFLD group exhibited lower education levels, lower smoking prevalence, and a higher proportion of never-drinkers (all P < 0.001). Regarding medical history, patients with MAFLD had significantly higher prevalences of hypertension (37% vs. 16%), cerebrovascular disease (7.7% vs. 3.2%), and heart disease (12% vs. 6.7%) (all P < 0.001).

Anthropometrically, MAFLD patients demonstrated higher weight, waist circumference, and BMI (all P < 0.001). Laboratory analyses revealed that the MAFLD group had significantly higher levels of FPG, HbA1c, TG, and SBP, along with lower HDL-C levels (all P < 0.05).

Regarding insulin resistance indices, all evaluated markers differed significantly between groups. MAFLD participants exhibited higher TyG, TyG-BMI, TyG-WC, TyG-WHtR, CVAI, and METS-IR, whereas SPISE was significantly lower in the MAFLD group (all P < 0.001).

### 3.2 Association of insulin resistance indices with MAFLD

Multivariable logistic regression analyses were performed to evaluate the independent associations between each insulin resistance index and MAFLD after sequential adjustment for potential confounders. The results from the fully adjusted models (Model 3) are presented in **Table 2**.

**Table 2:** Association of insulin resistance indices with MAFLD in multivariable logistic regression models.

Model	IR_Index	OR_CI	P_value
ModelA (+TyG)	TyG	2.07 (1.79-2.39)	<0.001
ModelB (+TyG_BMI)	TyG_BMI	1.02 (1.02-1.02)	<0.001
ModelC (+TyG_WC)	TyG_WC	1.01 (1.01-1.01)	<0.001
ModelD (+TyG_WHtR)	TyG_WHtR	2.99 (2.57-3.47)	<0.001
ModelE (+SPISE)	SPISE	0.57 (0.53-0.61)	<0.001
ModelF (+METS_IR)	METS_IR	1.11 (1.09-1.13)	<0.001
ModelG (+CVAI)	CVAI	1.02 (1.02-1.03)	<0.001

In the fully adjusted model, all evaluated insulin resistance indices demonstrated statistically significant associations with MAFLD (all P < 0.001). Higher levels of TyG, TyG-BMI, TyG-WC, TyG-WHtR, METS-IR, and CVAI were each independently associated with increased odds of MAFLD. Specifically, the odds ratios (ORs) per unit increase were 2.07 (95% CI: 1.79–2.39) for TyG, 1.02 (95% CI: 1.02–1.02) for TyG-BMI, 1.01 (95% CI: 1.01–1.01) for TyG-WC, 2.99 (95% CI: 2.57–3.47) for TyG-WHtR, 1.11 (95% CI: 1.09–1.13) for METS-IR, and 1.02 (95% CI: 1.02–1.03) for CVAI. In contrast, the SPISE index was inversely associated with MAFLD, with an OR of 0.57 (95% CI: 0.53–0.61) per unit increase.

Notably, TyG-WHtR exhibited the strongest magnitude of association among the indices examined, while TyG-BMI, TyG-WC, and CVAI showed relatively modest ORs, reflecting differences in their unit scales and distributions. These findings indicate that all evaluated insulin resistance markers are independently associated with MAFLD in Chinese adults with type 2 diabetes, albeit with varying effect sizes.

### 3.3 Diagnostic performance of insulin resistance indices for MAFLD

**Figure 1:**
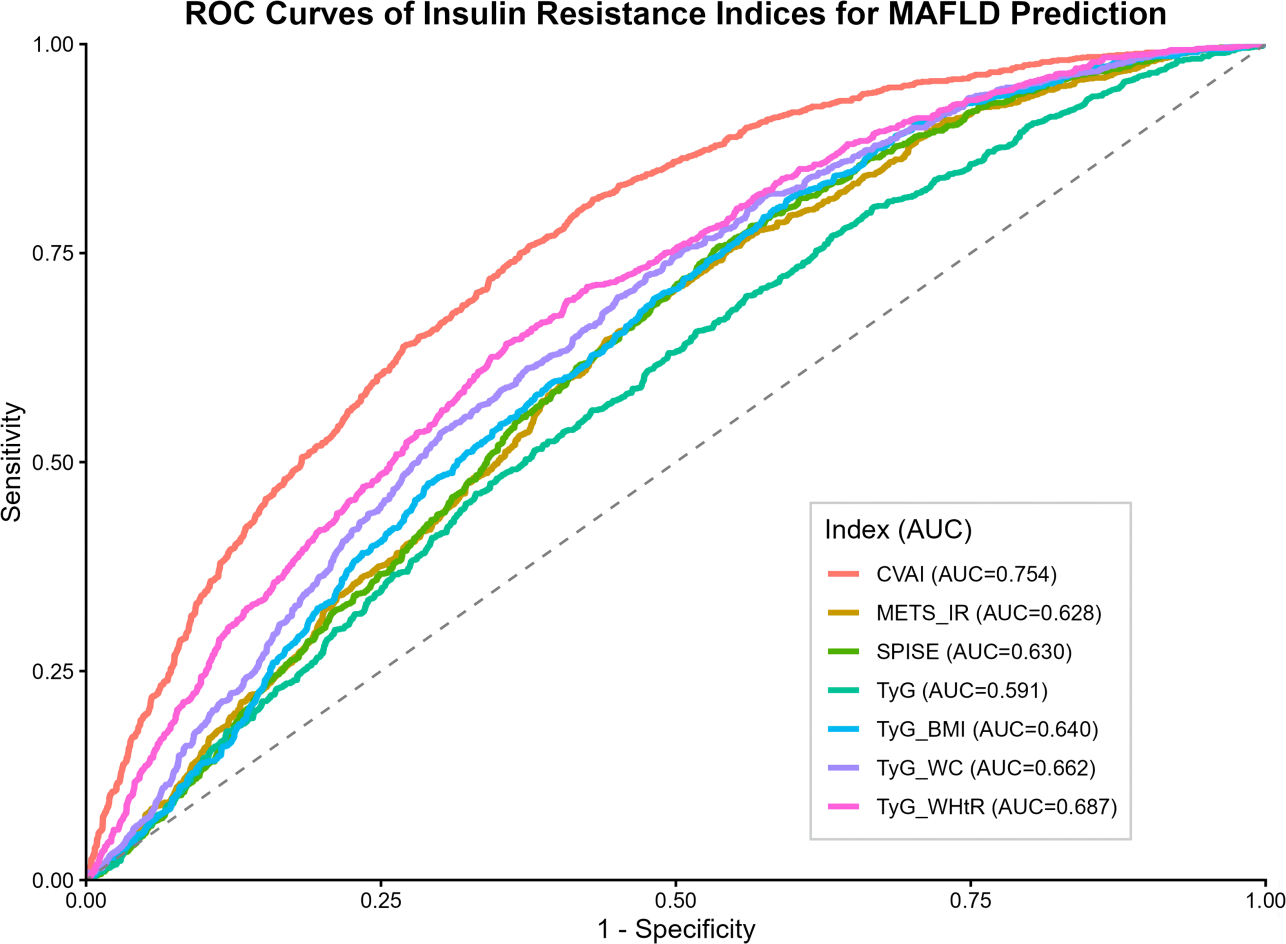
Receiver operating characteristic (ROC) curves of seven insulin resistance indices (TyG, TyG‑BMI, TyG‑WC, TyG‑WHtR, CVAI, SPISE, and METS‑IR) for predicting MAFLD in Chinese adults with type 2 diabetes.

ROC curve analysis was performed to evaluate and compare the discriminative ability of each insulin resistance index for identifying MAFLD. The AUC, optimal cut-off values determined by the Youden index, and corresponding sensitivity and specificity are summarized in **Table 3**, and the ROC curves are presented in **Figure 1**.

**Table 3:** Diagnostic performance of insulin resistance indices for MAFLD.

Index	AUC (95% CI)	Cutoff	Sensitivity	Specificity	P value (vs CVAI)
TyG	0.591 (0.571-0.611)	9.024	0.698	0.441	<0.001
TyG_BMI	0.640 (0.620-0.659)	211.909	0.813	0.406	<0.001
TyG_WC	0.662 (0.643-0.682)	786.937	0.754	0.494	<0.001
TyG_WHtR	0.687 (0.668-0.706)	5.022	0.693	0.593	<0.001
CVAI	0.754 (0.737-0.771)	114.146	0.814	0.571	1.000
SPISE	0.630 (0.610-0.650)	6.642	0.758	0.464	<0.001
METS_IR	0.628 (0.608-0.648)	37.145	0.753	0.457	<0.001

Among all evaluated indices, CVAI exhibited the highest discriminative ability, with an AUC of 0.754 (95% CI: 0.737–0.771). The optimal cut-off value for CVAI was 114.146, yielding a sensitivity of 81.4% and a specificity of 57.1%. TyG-WHtR demonstrated the second-highest AUC of 0.687 (95% CI: 0.668–0.706), followed by TyG-WC (AUC = 0.662, 95% CI: 0.643–0.682) and TyG-BMI (AUC = 0.640, 95% CI: 0.620–0.659). SPISE and METS-IR showed moderate discriminative performance, with AUCs of 0.630 (95% CI: 0.610–0.650) and 0.628 (95% CI: 0.608–0.648), respectively. The TyG index alone had the lowest AUC of 0.591 (95% CI: 0.571–0.611).

Pairwise comparisons using DeLong’s test revealed that the AUC of CVAI was significantly higher than that of all other insulin resistance indices examined (all P < 0.001). These findings indicate that CVAI possesses superior diagnostic performance for identifying MAFLD in Chinese adults with type 2 diabetes compared with traditional and composite insulin resistance markers.

### 3.4 Reclassification improvement for MAFLD diagnosis

**Figure 2:**
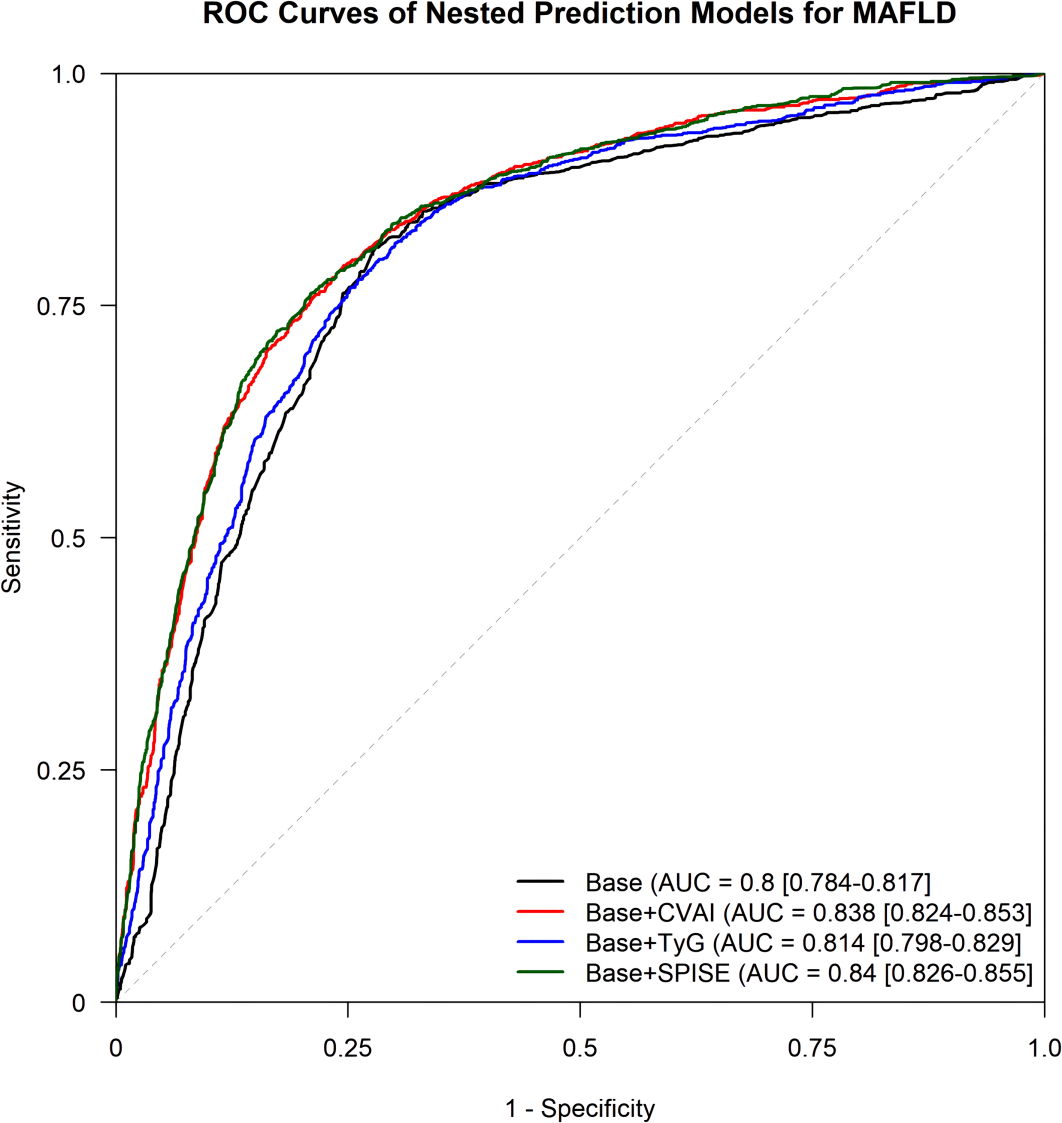
ROC curves of four nested prediction models for MAFLD.

**Figure 2** presents the ROC curves of the four nested discriminative models for MAFLD. The baseline model, which included age, sex, smoking, alcohol consumption, physical activity, and hypertension, yielded an AUC of 0.800 (95% CI: 0.784–0.817). Adding TyG to the baseline model increased the AUC to 0.814 (95% CI: 0.798–0.829). Notably, the models incorporating CVAI and SPISE demonstrated substantially improved discriminative ability, with AUCs of 0.838 (95% CI: 0.824–0.853) and 0.840 (95% CI: 0.826–0.855), respectively. These findings indicate that both CVAI and SPISE enhance the diagnostic performance of the baseline model, with SPISE showing a marginally higher AUC.

To further evaluate whether the addition of these insulin resistance indices could improve risk reclassification beyond the baseline model, we calculated the continuous NRI and IDI. The results of five pairwise comparisons are summarized in **Table 4**.

**Table 4:** Reclassification improvement of adding IR indices to baseline model.

Comparison	NRI (95% CI)	P (NRI)	IDI (95% CI)	P (IDI)
Base + CVAI vs Base	0.598 (0.533-0.665)	<0.001	0.081 (0.072-0.090)	<0.001
Base + TyG vs Base	0.411 (0.342-0.481)	<0.001	0.031 (0.025-0.037)	<0.001
Base + SPISE vs Base	0.574 (0.501-0.644)	<0.001	0.087 (0.076-0.097)	<0.001
Base+CVAI vs Base+TyG	0.397 (0.326-0.471)	<0.001	0.049 (0.040-0.059)	<0.001
Base+CVAI vs Base+SPISE	-0.116 (-0.189--0.045)	<0.001	-0.006 (-0.012-0.000)	0.052

As shown in **Table 4**, adding CVAI, TyG, or SPISE to the baseline model all significantly improved risk reclassification. The NRI and IDI for adding CVAI were 0.598 (95% CI: 0.533–0.665, P < 0.001) and 0.081 (95% CI: 0.072–0.090, P < 0.001), respectively. For TyG, the NRI was 0.411 (95% CI: 0.342–0.481, P < 0.001) and IDI was 0.031 (95% CI: 0.025–0.037, P < 0.001). For SPISE, the NRI was 0.574 (95% CI: 0.501–0.644, P < 0.001) and IDI was 0.087 (95% CI: 0.076–0.097, P < 0.001). These findings indicate that each index independently provides incremental discriminative value over the baseline clinical model.

When directly comparing the CVAI-enhanced model against the TyG-enhanced model, CVAI still demonstrated significant improvement (NRI = 0.397, 95% CI: 0.326–0.471, P < 0.001; IDI = 0.049, 95% CI: 0.040–0.059, P < 0.001). However, compared with the SPISE-enhanced model, CVAI did not show a significant improvement in discrimination: the NRI was –0.116 (95% CI: –0.189 to –0.045, P < 0.001) and the IDI was –0.006 (95% CI: –0.012 to 0.000, P = 0.052), suggesting that SPISE might be slightly superior in reclassification, though the IDI difference did not reach statistical significance.

### 3.5 Decision curve analysis for MAFLD prediction

**Figure 3:**
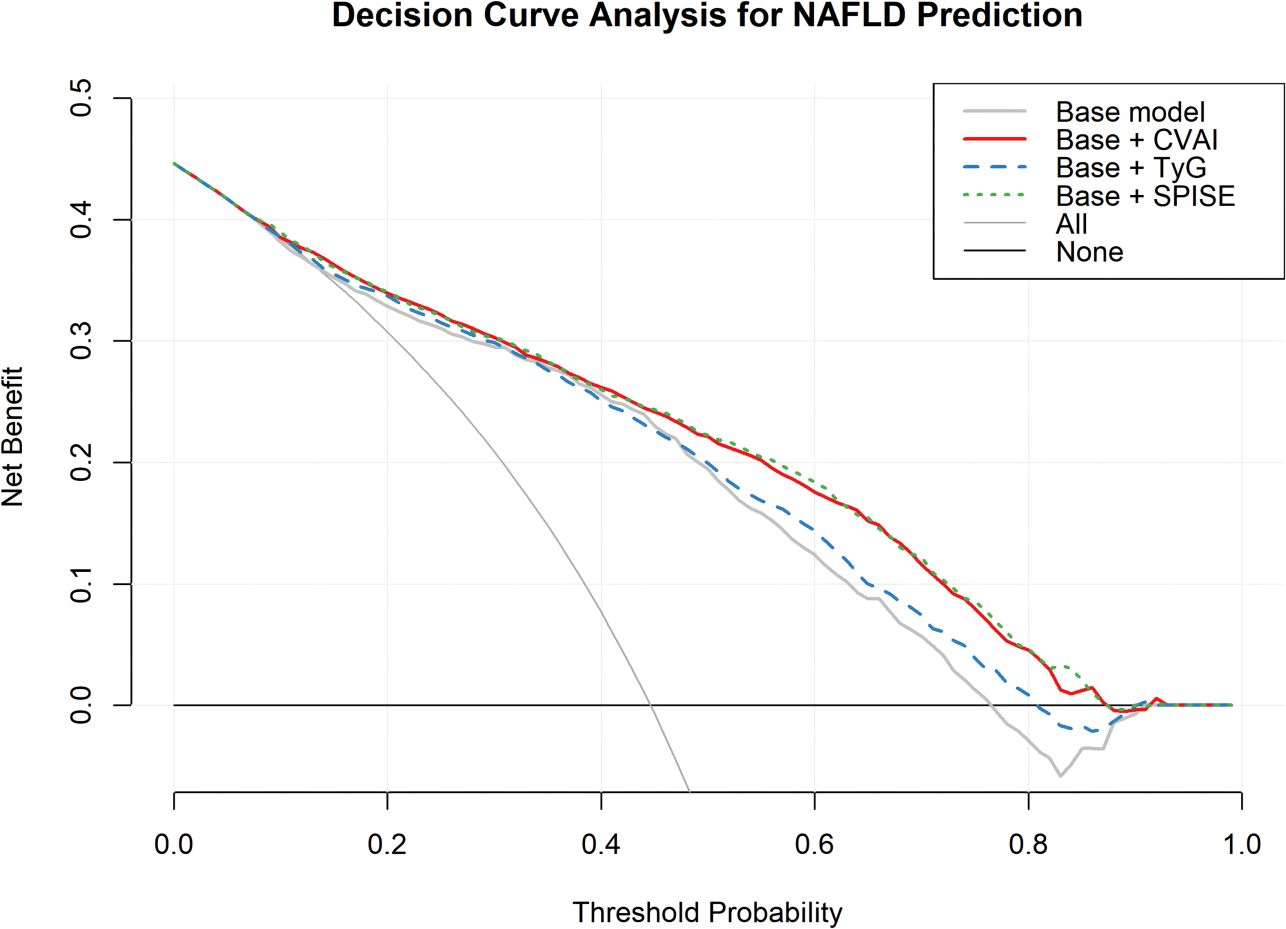
Decision curve analysis for MAFLD prediction.

To evaluate the clinical utility of incorporating different insulin resistance indices into MAFLD risk prediction, decision curve analysis was performed for four models: the baseline model and models additionally incorporating CVAI, TyG, or SPISE. The net benefits of these models across a range of threshold probabilities are presented in **Figure 3**.

The baseline model demonstrated positive net benefit across threshold probabilities ranging from approximately 10% to 75%. The addition of TyG extended the range of clinical utility to threshold probabilities between 10% and 80%. Notably, both the baseline + SPISE and baseline + CVAI models exhibited the broadest ranges of clinical applicability, with positive net benefits observed across threshold probabilities from 10% to 90%.

Throughout the majority of threshold probability ranges, the baseline + CVAI model consistently yielded the highest net benefit compared with the baseline model and models incorporating TyG. These findings suggest that incorporating CVAI into clinical risk prediction models enhances their net benefit across a wider and more clinically relevant range of threshold probabilities, supporting its potential utility as a decision-making tool for MAFLD risk assessment in patients with type 2 diabetes.

### 3.6 Non-linear relationship between CVAI and MAFLD risk

**Figure 4:**
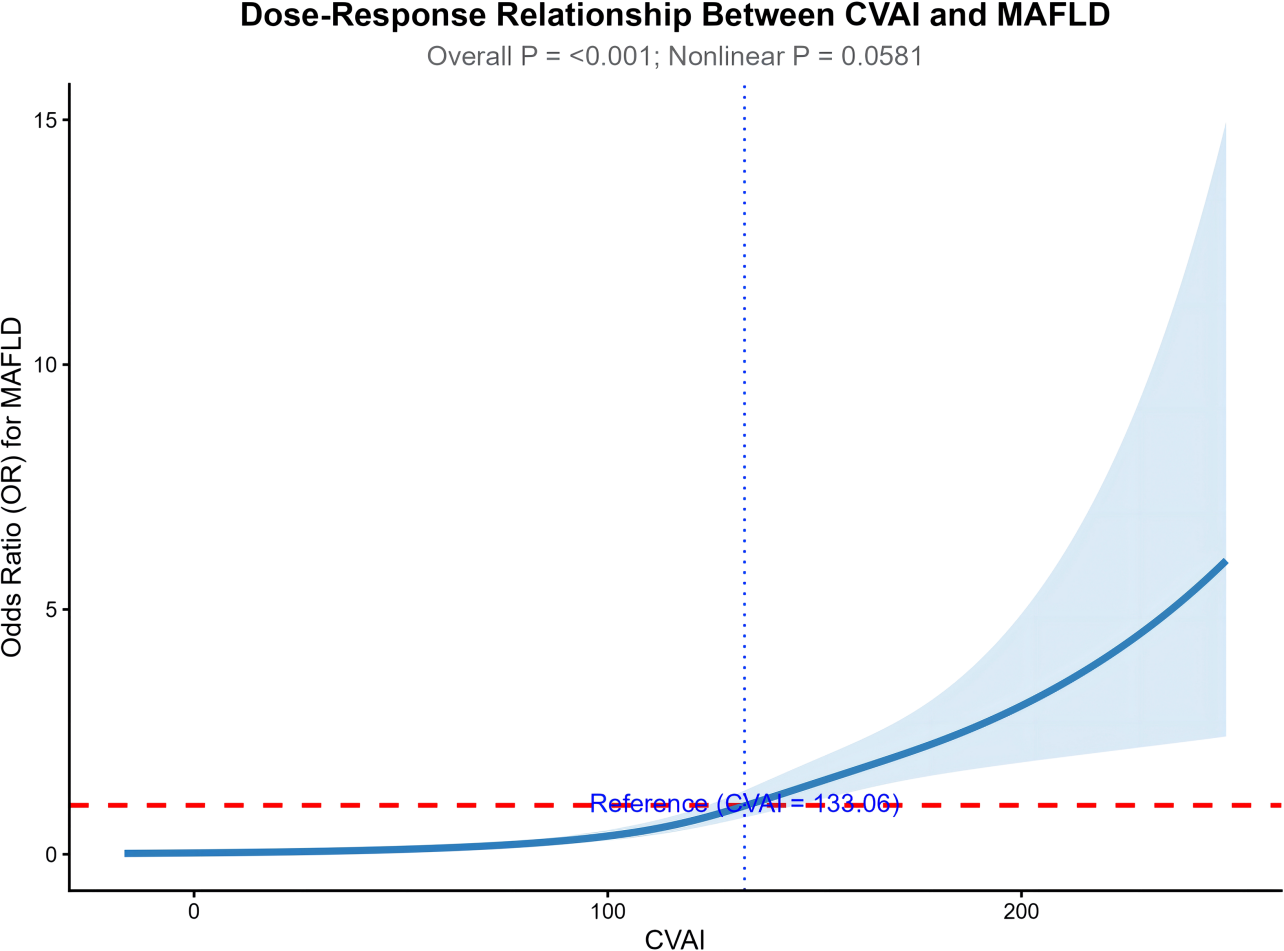
Restricted cubic spline (RCS) analysis showing the dose‑response relationship between CVAI and MAFLD risk.

To explore the potential non-linear relationship between CVAI and MAFLD risk, restricted cubic spline regression with four knots placed at the 5th, 35th, 65th, and 95th percentiles of the CVAI distribution was fitted within the fully adjusted logistic regression model. The results are visualized in **Figure 4**, with the reference value set at the median CVAI level (133.06).

The RCS analysis revealed a significant overall association between CVAI and MAFLD risk (overall P < 0.001). The test for non-linearity yielded a P value of 0.0581, suggesting a trend toward a non-linear relationship that approached but did not reach conventional statistical significance. As illustrated in **Figure 4**, the odds ratio for MAFLD increased progressively with rising CVAI levels. The risk curve exhibited a relatively steeper slope at higher CVAI values, indicating a potential threshold effect where the risk of MAFLD accelerates beyond a certain CVAI level.

These findings suggest that CVAI is positively associated with MAFLD risk in a predominantly linear manner, although a subtle non-linear trend may exist at the upper range of the CVAI distribution.

### 3.7 Subgroup analysis and interaction for the CVAI–MAFLD association

**Figure 5:**
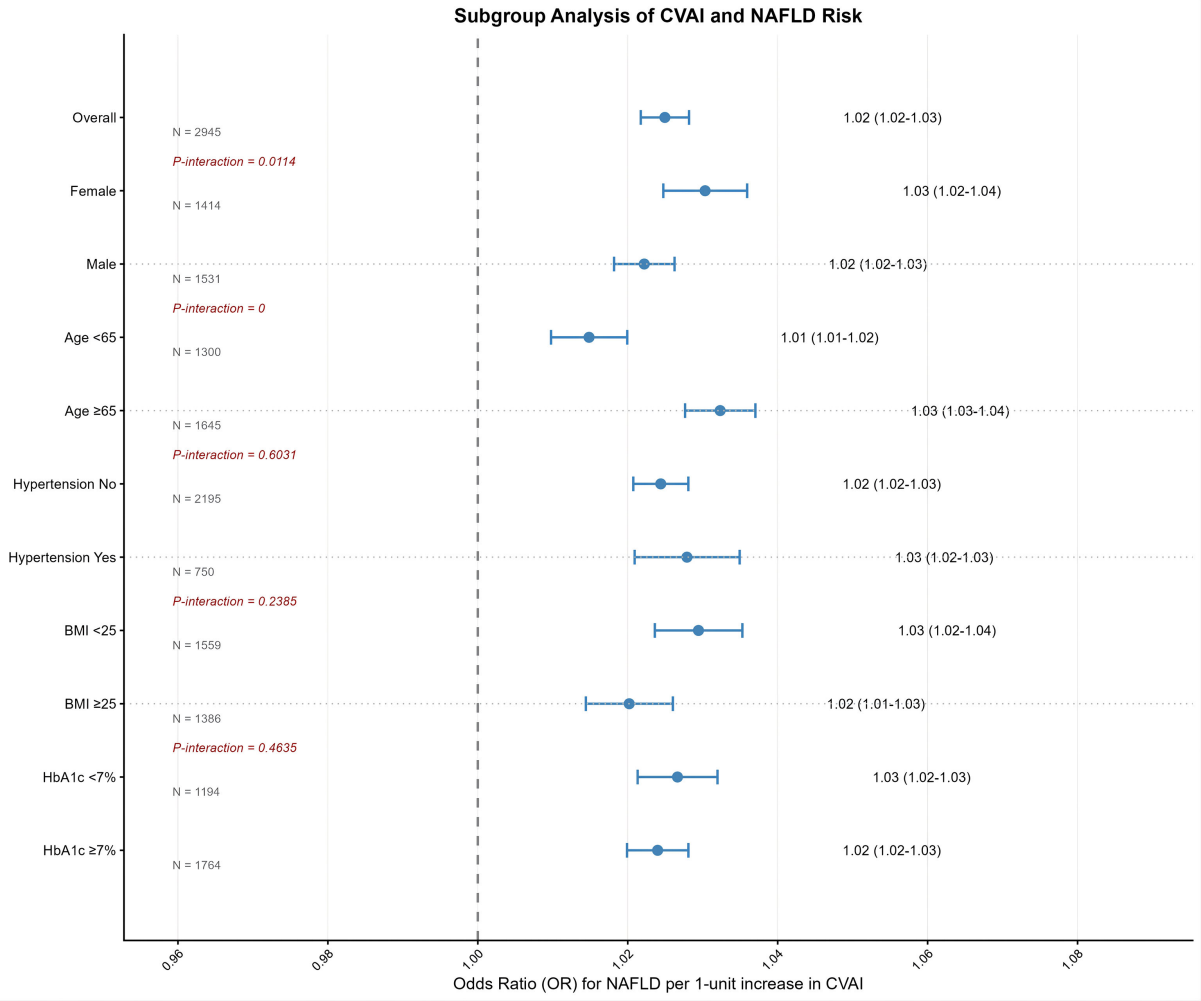
Forest plot of subgroup analyses for the association between CVAI and MAFLD, stratified by sex, age, hypertension, BMI, and HbA1c.

To assess the consistency of the association between CVAI and MAFLD across different clinical and demographic subgroups, we performed stratified analyses by sex, age, hypertension status, BMI, and HbA1c level. The fully adjusted ORs for MAFLD per unit increase in CVAI within each subgroup, along with interaction P values, are presented in **Figure 5**.

The positive association between CVAI and MAFLD was consistently observed across all subgroups examined. However, significant interactions were identified for sex and age. The association was significantly stronger in females (OR = 1.030, 95% CI: 1.025–1.036) than in males (OR = 1.022, 95% CI: 1.018–1.026), with a P for interaction of 0.011. Regarding age, the association was markedly stronger in participants aged ≥65 years (OR = 1.032, 95% CI: 1.028–1.037) than in those aged <65 years (OR = 1.015, 95% CI: 1.010–1.020), yielding a highly significant interaction (P for interaction = 9.14 × 10^-9^).

In contrast, no significant interactions were observed for hypertension status, BMI category, or HbA1c level. The ORs for MAFLD per unit increase in CVAI were comparable between participants with and without hypertension (OR = 1.028 vs. 1.024; P for interaction = 0.603), between those with BMI ≥25 kg/m² and <25 kg/m² (OR = 1.020 vs. 1.029; P for interaction = 0.239), and between those with HbA1c ≥7% and <7% (OR = 1.024 vs. 1.027; P for interaction = 0.463).

These findings indicate that the association between CVAI and MAFLD is robust across various patient subgroups, with significantly stronger associations observed in females and older adults.

## 4 Discussion

This study comprehensively evaluated and compared the discriminative ability of CVAI, TyG, TyG-BMI, TyG-WC, TyG-WHtR, METS-IR, and SPISE for MAFLD in a large community-based cohort of Chinese adults with type 2 diabetes. The main findings are fourfold. First, all evaluated insulin resistance indices were independently associated with MAFLD after full adjustment for confounders, with TyG-WHtR exhibiting the strongest magnitude of association. Second, among all single indices, CVAI demonstrated the highest discriminative ability for MAFLD (AUC = 0.754), significantly outperforming TyG, TyG-BMI, TyG-WC, TyG-WHtR, METS-IR, and SPISE. Third, adding CVAI to a baseline clinical model significantly improved risk reclassification (NRI = 0.598, IDI = 0.081) and yielded the broadest clinical utility across threshold probabilities from 10% to 90% in decision curve analysis. Fourth, the association between CVAI and MAFLD was robust across subgroups, with significantly stronger effects observed in females and older adults (both P for interaction < 0.05). These findings suggest that CVAI, as a marker of visceral adipose dysfunction, may serve as a superior non-invasive tool for identifying MAFLD in Chinese patients with T2DM.

The positive association between CVAI and MAFLD observed in our study aligns with accumulating evidence supporting the role of visceral adiposity in the pathogenesis of fatty liver disease. Chen et al. demonstrated that CVAI was more strongly associated with NAFLD prevalence than were VAI, lipid accumulation product, and waist circumference in the general Chinese population (27). Subsequent studies extended these findings to specific populations. Tang et al. reported a positive correlation between CVAI and MAFLD in Chinese adults with T2DM, although their analysis was limited by a relatively small sample size and did not comprehensively compare CVAI with other IR indices (33). Niu et al. further validated the diagnostic value of CVAI for MAFLD and elevated ALT in non-obese Chinese adults, highlighting its applicability beyond obese populations (34). Our study builds upon these prior investigations by systematically comparing CVAI against a panel of established IR indices in a large T2DM cohort, thereby providing robust evidence that CVAI possesses superior diagnostic performance even after accounting for traditional metabolic risk factors.

The superior discriminative ability of CVAI (AUC = 0.754) in our study is consistent with its strong correlation with visceral fat area (VFA). Previous research demonstrated that CVAI correlates more strongly with CT-measured VFA (*r* = 0.755) than does VAI (*r* = 0.417), indicating that CVAI is a more reliable surrogate for visceral adiposity in Chinese populations (24). Although our study did not include direct VFA measurement, this strong external evidence supports CVAI as a validated surrogate for visceral adiposity. Future studies should directly measure VFA (e.g., by CT or MRI) within the same cohort to further confirm this relationship. Given that visceral fat accumulation is a key driver of insulin resistance, systemic inflammation, and hepatic steatosis, indices that accurately reflect visceral adiposity are expected to exhibit stronger associations with MAFLD (35, 36). Indeed, a biopsy-proven study by Li et al. revealed that CVAI was independently associated with the severity of hepatic steatosis and fibrosis in NAFLD patients, further supporting its pathophysiological relevance (37). The consistency of these findings across different study designs and populations underscores the robustness of CVAI as a biomarker for fatty liver disease.

Although TyG and its derivatives (TyG-BMI, TyG-WC, TyG-WHtR) have been widely validated as surrogate markers of insulin resistance and shown discriminative value for MAFLD (18–20), our study reveals that CVAI outperforms these indices in terms of diagnostic accuracy. Several factors may explain this discrepancy. First, TyG-based indices primarily capture glucose–lipid metabolic disorders but do not directly quantify adipose tissue distribution or function. In contrast, CVAI integrates age, BMI, waist circumference, and lipid profiles to specifically estimate visceral adipose dysfunction, which is more closely linked to the pathogenesis of MAFLD (30). Second, the strong linear correlation between CVAI and VFA enables it to capture the cumulative burden of visceral adiposity, whereas TyG indices may reflect more transient metabolic fluctuations (33). Third, the inclusion of age in the CVAI formula may partially account for the age-related decline in metabolic reserve and increase in visceral fat accumulation, which could explain its particularly strong performance in older adults observed in our subgroup analysis.

METS-IR and SPISE showed moderate discriminative ability in our study (AUC = 0.628 and 0.630, respectively), consistent with previous reports. METS-IR, which integrates fasting glucose, triglycerides, and BMI, has been proposed as a simple tool for assessing insulin resistance in non-obese populations (21). However, its performance in T2DM patients with MAFLD appears inferior to that of CVAI, possibly because METS-IR does not account for waist circumference or HDL-C—both critical determinants of visceral adiposity and metabolic health. SPISE, originally developed as an insulin sensitivity estimator for adolescents, demonstrated an inverse association with MAFLD (OR = 0.57), reflecting its role as a protective factor. Interestingly, SPISE showed an AUC comparable to that of CVAI when added to the baseline model (0.840 vs. 0.838), and the NRI for CVAI vs. SPISE was negative (–0.116), suggesting that SPISE may have slight advantages in reclassification. However, the IDI difference did not reach statistical significance (P = 0.052), indicating that the two indices are largely comparable in terms of discrimination improvement.

A notable finding of our study is the significant interaction between CVAI and both sex and age. The association between CVAI and MAFLD was significantly stronger in females (OR = 1.030) than in males (OR = 1.022), with a P for interaction of 0.011. This sex-specific pattern has been observed in previous studies. Ma et al. reported that the TG/HDL-C ratio, another IR-related index, exhibited a stronger association with MAFLD in females than in males (38). Similarly, Fan et al. demonstrated that the TG/HDL-C ratio had better diagnostic ability for NAFLD in Chinese females (39). The underlying mechanisms may relate to the protective effects of estrogen on hepatic lipid metabolism, which diminish after menopause, leading to accelerated visceral fat accumulation and metabolic deterioration in postmenopausal women (40). Given that the mean age of female participants in our study was 64 years, the majority were likely postmenopausal, which could explain their heightened susceptibility to visceral adiposity–related MAFLD risk.

Regarding age, the association between CVAI and MAFLD was markedly stronger in participants aged ≥65 years (OR = 1.032) than in those <65 years (OR = 1.015), with a highly significant interaction (P = 9.14 × 10^-9^). Aging is accompanied by progressive accumulation of visceral fat—even in the absence of weight gain—due to hormonal changes and decreased physical activity (41). Moreover, age-related decline in mitochondrial function and increased oxidative stress may render older adults more vulnerable to the deleterious effects of visceral adiposity on hepatic metabolism. These findings underscore the importance of considering age and sex when interpreting CVAI for MAFLD risk assessment and suggest that CVAI may be particularly useful for identifying high-risk individuals among older adults and postmenopausal women.

The findings of this study have several important clinical implications. First, CVAI, as a simple and non-invasive index derived from routine clinical parameters, could serve as an effective screening tool for MAFLD in T2DM patients, a high-risk population with limited access to liver biopsy or advanced imaging techniques. The optimal cut-off value of 114.146 identified in our study (sensitivity 81.4%, specificity 57.1%) provides a practical threshold for clinical decision-making. Second, the superior discriminative ability of CVAI compared with traditional IR indices suggests that assessing visceral adiposity may be more relevant than measuring global insulin resistance for MAFLD risk stratification in T2DM. This aligns with the growing recognition that adipose tissue dysfunction—rather than adiposity per se—is the key driver of metabolic complications (48). Third, the significant improvement in reclassification and net benefit demonstrated by CVAI in decision curve analysis supports its utility for guiding clinical management. Patients with elevated CVAI may benefit from more intensive lifestyle interventions targeting visceral fat reduction, such as calorie restriction and increased physical activity, which have been shown to improve both metabolic health and liver histology (35). Given the stronger association observed in females and older adults (≥65 years), CVAI-based screening may be particularly valuable for targeted risk assessment in postmenopausal women and elderly individuals.

The strong association between CVAI and MAFLD can be explained by the central role of visceral adipose tissue in the pathogenesis of insulin resistance and hepatic steatosis. Visceral adiposity promotes the release of free fatty acids into the portal circulation, leading to increased hepatic uptake and triglyceride accumulation (42). Simultaneously, dysfunctional adipose tissue secretes pro-inflammatory adipokines (e.g., TNF-α, IL-6) and reduces the secretion of protective adipokines (e.g., adiponectin), contributing to systemic inflammation and hepatic insulin resistance (43, 44). These processes activate hepatic *de novo* lipogenesis via SREBP-1c and ChREBP, while inhibiting fatty acid oxidation, thereby exacerbating lipid deposition and triggering a vicious cycle of metabolic dysregulation (45, 46).

Recent single-cell RNA sequencing studies have provided deeper insights into the hepatic microenvironment under diabetic conditions. Dai et al. demonstrated that high glucose and high fat conditions induce pathological changes in hepatocytes, including overexpression of TXNIP leading to oxidative stress, activation of hepatic stellate cells via HIC5, and capillarization of liver sinusoidal endothelial cells associated with FABP4 elevation (47). These findings highlight the interplay between glucose toxicity, lipotoxicity, and cellular stress in driving liver pathology. CVAI, by integrating parameters that reflect both adiposity (BMI, WC) and metabolic dysfunction (TG, HDL-C, age), may capture the cumulative burden of these interconnected pathways, thereby offering a more comprehensive assessment of MAFLD risk than indices focused solely on glucose or lipid metabolism (48).

This study has several strengths. It included 2,945 participants, representing a relatively large sample for systematically comparing CVAI with multiple IR indices for MAFLD diagnostic assessment in a T2DM cohort. The large sample size (n = 2,945) enabled robust multivariable adjustment and subgroup analyses. The comprehensive analytical approach—including logistic regression, ROC curves with DeLong tests, NRI/IDI, RCS, DCA, and subgroup analyses—provides a holistic evaluation of diagnostic performance. The use of a community-based sample enhances the generalizability of findings to real-world clinical settings. Furthermore, the inclusion of external validation references (e.g., biopsy-proven studies, CT-measured VFA correlations) strengthens the biological plausibility of our findings (37, 41).

However, several limitations must be acknowledged. First, the cross-sectional design precludes causal inferences. Although we identified strong associations between CVAI and MAFLD, prospective studies are needed to determine whether CVAI predicts incident MAFLD or disease progression over time. Second, hepatic steatosis was diagnosed by abdominal ultrasound rather than liver biopsy or MRI-PDFF, which may have led to misclassification of mild steatosis. Although ultrasound is widely used in clinical practice and has acceptable sensitivity for moderate-to-severe steatosis, it may underestimate mild cases (49). Future studies incorporating controlled attenuation parameter (CAP) or MRI-PDFF could provide more accurate assessments. Third, the single-center design and exclusive focus on Chinese adults may limit generalizability to other ethnic populations. However, recent studies have demonstrated that CVAI also shows promising predictive value for MASLD in American populations, suggesting potential cross-ethnic applicability (50). Fourth, we did not assess longitudinal changes in CVAI or its response to interventions, which could provide insights into its utility for monitoring treatment efficacy. Fifth, we did not collect data on dietary patterns (e.g., fructose intake), sleep quality/duration, or detailed medication history (e.g., statins, steroids), which may have introduced residual confounding. Sixth, we did not directly measure visceral fat area (VFA) using CT or MRI. The mechanistic interpretation linking CVAI to visceral adiposity therefore relies on external validation rather than internal data. Future studies should directly quantify VFA using CT or MRI in T2DM cohorts to validate the correlation with CVAI and to explore the mediating roles of inflammatory cytokines (e.g., IL-6, TNF-α) and liver fibrosis markers (e.g., FIB-4, NFS).

## 5 Conclusion

This study demonstrates that CVAI is independently associated with MAFLD and exhibits superior diagnostic performance compared with traditional insulin resistance indices in Chinese adults with type 2 diabetes. The association is particularly strong in females and older adults, underscoring the need to consider demographic factors in risk stratification. CVAI significantly improves risk reclassification and demonstrates favorable clinical utility across a wide range of threshold probabilities. As a simple, non-invasive, and cost-effective index derived from routine clinical parameters, CVAI holds promise as a practical tool for MAFLD screening and risk assessment in T2DM populations. Prospective studies are warranted to validate its diagnostic utility for incident MAFLD, disease progression, and long-term outcomes.

## Data Availability

The data in this article are derived from electronic health records at community health service centers and contain sensitive patient information, thus are not directly accessible to the public. The data were obtained in a de-identified format and authorized by the Ethics Committee of Longhua District People’s Hospital for use in this study. Requests for data access should be directed to the corresponding author and must be accompanied by a formal data sharing agreement and approval from the aforementioned committee. Corresponding author email: 54574963@qq.com. Requests to access these datasets should be directed to 54574963@qq.com.
